# Endobronchial Ultrasound Access to Pulmonary Vasculature in Thoracic Malignancy

**DOI:** 10.3390/cancers17040616

**Published:** 2025-02-11

**Authors:** Evangelia Koukaki, Nektarios Anagnostopoulos, Aikaterini Bakiri, Stavroula Zaneli, Grigorios Stratakos

**Affiliations:** Interventional Pulmonology Unit, 1st Respiratory Department of National and Kapodistrian University of Athens, Sotiria Chest Diseases Hospital, 11527 Athens, Greece; aris.anag@yahoo.gr (N.A.); aik.bakiri@gmail.com (A.B.); stavzaneli@gmail.com (S.Z.); grstrat@hotmail.com (G.S.)

**Keywords:** endobronchial ultrasound (EBUS), T4 staging, pulmonary embolism, transvascular needle aspiration (TVNA), intravascular approach

## Abstract

Endobronchial ultrasound (EBUS) has evolved beyond conventional applications in mediastinal staging and central pulmonary tumor diagnosis. It encompasses the assessment of pulmonary vasculature in patients with thoracic malignancies. EBUS can visualize major vessels and allow assessment of pulmonary embolism, differential diagnosis of endovascular lesions, and T staging. Additionally, EBUS-guided transvascular needle aspiration (TVNA) has proven valuable for sampling lesions behind vessels and diagnosing conditions such as pulmonary artery sarcoma and tumor embolism, with low complication rates reported. The PubMed and SCOPUS databases were searched up to November 2024 for articles in the English language reporting the use of EBUS for pulmonary vasculature assessment. References were also searched for relevant articles. The integration of EBUS with other modalities enhances staging and diagnostic capabilities in thoracic malignancies. Despite promising findings, limitations include suboptimal image quality and challenges in extensively assessing all the vasculature. Safety concerns, particularly with transvascular biopsy, remain minimal with expert handling, although further studies are needed to assess specific risks like hematogenous tumor seeding. EBUS continues to evolve, suggesting its potential to become the cornerstone in advanced thoracic diagnostics and treatment planning. This review systematically explores the feasibility, safety, and diagnostic utility of EBUS in pulmonary vasculature assessment, highlighting its potential as an indispensable tool in thoracic diagnostics and treatment planning.

## Simple Summary

Endobronchial ultrasound (EBUS) is currently used for far more than just diagnosing and staging lung cancer. It can also help physicians assess thoracic vasculature, such as the aorta and pulmonary arteries. With EBUS we can identify pulmonary embolism or non-thrombotic vascular lesions and acquire trasnsvascular access to lung tumors, while even intravascular lesions like artery sarcoma become approachable. Although EBUS is generally safe, related to very rare complications, more research is needed to confirm its reliability for thoracic vasculature exploration and to identify potential risks.

## 1. Introduction

The role of endobronchial ultrasound (EBUS) in thoracic malignancy is well established for mediastinal staging, access to left adrenal gland, and diagnosis of central pulmonary tumors. Beyond these applications, EBUS has emerged as a valuable tool in the evaluation of key thoracic vessels. There is an increasing bibliography of cases concerning the use of EBUS for the exploration of pulmonary vasculature, aortic arch, and its branches. In fact, assessing or transversing a vessel in order to obtain tissue samples is nowadays more of a technique than a complication.

Traditionally, vascular involvement in thoracic malignancies has been assessed using advanced imaging modalities such as computed tomography (CT) or magnetic resonance imaging (MRI). The main medical indications in which EBUS could target intrathoracic vessels include (a) pulmonary embolism, (b) non-thrombotic endovascular lesions or vascular tumors, (c) assessment of T4 status, and (d) transvascular endosonographic needle aspiration of a lesion behind a vessel. Additionally, EBUS provides the unique advantage of real-time visualization and the possibility of minimally invasive tissue sampling simultaneously.

To our knowledge there is no review of the literature focusing on the use of EBUS to assess the vessels in a systematic approach in patients with thoracic malignancy. The PubMed and SCOPUS databases were searched for articles in the English language reporting the use of EBUS for pulmonary embolism, non-thrombotic vascular lesions or vascular tumors, transvascular needle aspiration, and T4 staging. We found a total of 118 results in PubMed and 107 in Scopus. The search was performed up to November 2024. No posters, oral presentations from conferences, or book chapters were included. After removal of duplicates, non-related articles were excluded based on their title and abstract. The full text of the remaining articles was studied, and their references were also searched for relevant articles. The results are discussed in the manuscript and summarized in Table 1, Table 2, Table 3 and Table 4.

This review systematically examines the current published evidence on feasibility, safety, and efficacy of the EBUS approach to the pulmonary vasculature and define indications, risk factors, and its expanding role in clinical practice.

## 2. Systematic Assessment of the Vasculature

EBUS offers a valuable approach for visualizing thoracic vessels. The blood vessels observable with EBUS include the aortic arch, ascending and descending aorta, left and right pulmonary artery (PA) brunches, azygos vein, superior vena cava, as well as pulmonary veins [1,2]. A structured systematic bronchoscopic approach has been proposed so far only for the pulmonary arteries assessment, but familiarization with the respective anatomical landmarks can allow for assessment of all the vessels adjacent to the main and lobar bronchi.

Aumiller et al. [3] were the first to propose a systematic methodology for thorough investigation of the pulmonary arteries by EBUS, aiming to assess sensitivity for pulmonary embolism diagnosis. In that way, they were able to adequately visualize the pulmonary trunk, the right and the left PA, as well as their proximal branches. Li P et al. as well as Juul et al. also described paths of exploration [4,5].

In comparison to traditional imaging techniques, that is to say CT or MRI, EBUS offers the advantage of real-time assessment without exposure to radiation. Apart from visualizing the vessels, there are scarce data regarding the assessment of the dimension of the vessels. Although there seems to exist a statistical correlation of EBUS and CT measurements of the left main artery and the ascending aorta, the limits of agreement are wide [6]. Thus, EBUS cannot be used for vessels’ dimension measurements interchangeably with CT.

The main limitation in evaluating vasculature are the peripheral vascular branches as they remain inaccessible via EBUS. Operator’s expertise for identifying anatomical landmarks and optimizing imaging is important for the identification of subtle vascular abnormalities.

## 3. Endovascular Lesions

### 3.1. Pulmonary Embolism

Pulmonary embolism (PE) is the most common vascular incidental finding, particularly in patients with malignancies probably given that cancer associated hypercoagulability increases thrombotic risk. Although, the gold standard for PE diagnosis is computed tomography pulmonary angiography (CTPA) with intravenous (iv) contrast enhancement, EBUS has demonstrated utility as an alternative diagnostic tool. To our knowledge the first report of a PE diagnosed with an EBUS bronchoscope was in 2008 by Casoni et al. [7] who confirmed a previously reported thrombus in a CTPA and differentiated it from a sarcoma based on its echomorphological characteristics (namely endovascular lesion floating and not attached to the arterial wall).

Aumillier et al. conducted the first study assessing the accuracy and safety of visualizing central PEs by EBUS in comparison to CTPA [3]. In this clinical pilot study, 32 patients with central PEs had 101 thrombi identified in their CTPA and subsequently were examined in a standardized way with EBUS. EBUS had 96% (97/101) accuracy in detection of the known thrombi and 100% diagnosis of PE was managed (by identifying at least one thrombus per case). The four emboli that were not detected included one in a middle lobe and three in a left upper lobe artery. As the authors state, “the most important limitation of EBUS is that it can only visualize central PEs”. It is noteworthy that the bronchoscopists performing in this study were moderately experienced. As more experience was gained, the medium duration per case was reduced from 5 min in the beginning of the study to 3 min in the later phase, suggesting that detecting PE with EBUS might not be a time-consuming skill to master. The limitation of this study was that the location of the thrombi was already known and in the case of absence of this data it is unsure whether the results would be the same.

Since this study, there have been several cases reported confirming a known PE [3,7,8,9,10,11,12,13], as well as several cases of PEs as incidental findings during EBUS examination [14,15,16,17,18,19,20,21,22,23,24,25,26,27,28,29,30,31,32] (Table 1). The estimated prevalence of incidental PE recognition in patients undergoing EBUS in whom the operator does not systematically assess the PAs is 0.7% [21]. In the majority of incidental PE findings, the patients had significant predisposing risk factors for thromboembolism, such as cancer.

In patients with suspected lung cancer undergoing EBUS, emerging evidence suggests that EBUS could serve as a screening tool for PE in this specific population. Some authors raise the question of whether great vessels should be examined for the presence of thrombi during the initial EBUS bronchoscopy examination for lung cancer staging [14] or whether there may be a role for this procedure in other specific populations [18]. Juul et al. demonstrated that systematic vascular assessment of the main PAs is feasible and efficient with a median time of only 2 min for the procedure and a positive predictive value of 100% [5]. The number needed to scan was 50 to detect one case of PE. The ultrasound findings that were considered indicative of PE are as follows: a visible hyperechoic area within a PA consistent with a thrombus or an area within the pulmonary area with absence of blood flow in color doppler examination [5]. Given the high risk of thrombotic complications in these patients, the favorable initial safety and effectiveness results should be seen with cautiousness.

Interestingly, Xing et al. [20] described the diagnosis of peripheral pulmonary embolism with the use of radial EBUS in a single patient with high clinical probability. This is the only report of radial EBUS interacting with the vasculature in such a way.

EBUS has also been used to differentiate hilar masses/extraluminal soft tissue from a clot in the pulmonary artery [11,33]. Only two cases of clots biopsy and histological confirmation of the presence of a thrombus exist [10,34]. EBUS has been helpful as a diagnostic approach for unstable patients in ICU [35,36,37,38] and with chronic thromboembolic pulmonary hypertension [39,40].

There is increasing literature regarding the possibility of thrombolysis of central PEs via transbronchial needle injection of rt-PA with the EBUS bronchoscope, which is still at an experimental level in porcine models [41,42]. The study was considered successful and raises questions on the potential future of EBUS-guided transbronchial thrombolysis as a feasible approach for centrally located pulmonary emboli. The authors comment that, in the future, EBUS could be useful for examining the extent of the thrombi and by determining the proximal extent of the disease, change its management, as well as confirm the successful outcome of an intervention.

In general, none of the patients in the literature developed complications due to EBUS bronchoscopy (Table 1). Contrary to the common belief that pulmonary embolism is a relative contraindication for bronchoscopy, there is increasing evidence for a favorable safety profile of this procedure in PE patients. In many cases, especially when patients are not able to undergo a CTPA examination due to renal insufficiency, allergy, pregnancy or hemodynamic instability, EBUS could provide an alternative for prompt diagnosis of PE which might be crucial for the patient. More studies are necessary to confirm this hypothesis.

Although EBUS bronchoscopy may not be able to become a standard diagnostic tool of the initial work-up for diagnosing a PE, the incidental finding of a PE could be of clinical importance for the patient’s well-being and should not be missed. Given that many patients who are candidates for EBUS are finally diagnosed with cancer, and thus have an increased risk for developing PE, early detection of such a complication could be substantial and lifesaving. Further research is warranted to elucidate if a PE finding alone may suffice to initiate anticoagulation therapy.

**Table 1:** Reported cases of pulmonary embolism diagnosed or confirmed with EBUS.

Authors	Year	N. of Cases	EBUS Indication	CTPA	Predisposing Factors (For Not Already Known PE Cases)
Casoni et al. [7]	2008	1	Visualization of known PE	Already shown PE	
Aumiller et al. [3]	2009	32	Visualization of known PE	Already shown PE	
Centikaya et al. [9]	2011	1	Visualization of known PE	Already shown PE	
Sentourk et al. [8]	2013	8	Visualization of known PE	Already shown PE	
Mahajan et al. [10]	2014	1	Visualization of known PE	Already shown PE	
Harris and Chaloub [11]	2014	1	Visualization of known PE	Already shown PE	
Evison et al. [12]	2015	1	Visualization of known PE	Already shown PE	
Chow et al. [13]	2021	1	Visualization of known PE	Already shown PE	
Sanz-Santos et al. [14]	2010	1	Incidental finding	Confirmed	Lung cancer
Swarz and Gillespie [15]	2011	1	Incidental finding	Confirmed	Lung cancer
Llopis Pastor [16]	2013	1	Incidental finding	Confirmed	Lung cancer
Sachdeva et al. [17]	2013	1	Incidental finding	Confirmed	Ankle injury
Segraves-Daniels [18]	2015	1	Incidental finding	Confirmed	Lung cancer
Goyal et al. [19]	2015	1	Incidental finding	Confirmed	None
Xing et al. [20]	2015	1	Incidental finding	Confirmed	Microscopic Polyangiitis
Erer et al. [21]	2016	4	Incidental finding	Confirmed	3/4 Lung cancer
Clay et al. [22]	2016	1	Incidental finding	Confirmed	Obesity, quadriparesis
Sariaydin et al. [23]	2016	1	Incidental finding	Confirmed	None
Torky et al. [24]	2018	1	Incidental finding	Confirmed	Lung cancer
Abuserewa [25]	2021	1	Incidental finding	Confirmed	Pericardial effusion of unknown aetiology—Diagnosis not mentioned
Abboud et al. [26]	2021	1	Incidental finding	Previous CTPA was given negative, but after EBUS rechecked and segmental PE was identified	Lung cancer, right internal jugular vein thrombosis
Fantin et al. [27]	2021	2	Incidental finding	Confirmed	Lung cancer
Solis Garcia et al. [28]	2022	1	Incidental finding of Second Episode of PE	Confirmed	Lung cancer
Subedi et al. [29]	2022	1	Incidental finding	Confirmed	Lung cancer
Maeda et al. [30]	2023	1	Incidental finding	Confirmed	Trauma, lung cancer
Bugalho et al. [31]	2024	1	Incidental finding of Second Episode of PE	Confirmed	Lung cancer, pulmonary hypertension
Cascon-Hernandez et al. [32]	2024	1	Incidental finding	Confirmed	Lung cancer
Park et al. [34]	2011	1	Diagnostic approach of endovascular lesion	Not performed	None
Joshi et al. [36]	2015	1	Diagnostic approach for unstable patient in ICU	Confirmed	COPD, possible cancer
Channick et al. [37]	2019	1	Diagnostic approach for unstable patient in ICU	Not performed	ICU stay, Surgery
Bertini et al. [38]	2020	1	Diagnostic approach for unstable patient in ICU	Not performed	Amyotrophic lateral sclerosis
Dhillon and Harris [39]	2016	1	Diagnostic approach for chronic pulmonary artery thrombosis	Not visualized in previous CT	Lung cancer
Decavele et al. [35]	2020	11	Assessment of PE in v-v ECMO SARS-CoV 2 patients	Confirmed in 9/10 of positive EBUS—Not confirmed in 0/1 of negative EBUS	SARS-CoV2
Juul et al. [5]	2024	100	Assessment of PE in possible NSCLC patients undergoing EBUS	Confirmed with CTPA	Lung cancer

### 3.2. Tumor Embolism

An abnormality within the vessel is not always a clot. It is a challenge to distinguish a thrombus from a non-thrombotic lesion such as a tumor embolism or a primary vascular neoplasm. Tumor embolism, a rare but significant condition, involves tumor cells lodging in vessels, often mimicking PE on imaging. The radiologic characteristics on CT or positron emission tomography (PET) are not able to differentiate between them and histology is often needed. Metastases in pulmonary arteries (also known as tumor embolism) were first described in 1897 by Schmidt [33] in autopsy studies. Since then, most of the reports of tumor embolism were made in autopsies as well.

The first EBUS-TBNA observation of tumor embolism was by Blanc et al. [43] in a patient with a history of thyroid cancer. Tumor embolism was suspected and confirmed after taking biopsy of an alleged pulmonary embolism not improving with extended anticoagulation for over a year. Since then, several cases of EBUS-TBNA from tumor embolism have been described [43,44,45,46,47,48,49,50,51] (Table 2). To our knowledge, nine cases of tumor embolism have been diagnosed with the use of EBUS: renal synovial carcinomas (two cases), thyroid cancer, melanoma, primary retroperitoneal synovial sarcoma, renal cell carcinoma, leiomyosarkoma, hepatocellular carcinoma, and colorectal carcinoma [43,44,45,46,47,48,49,50,51].

**Table 2:** Reported cases of non-thrombotic endovascular lesions diagnosed or reported with EBUS.

Author	Year	N. of Cases	EBUS Indication	Location	Biopsy of Intra Vascular Lesion	Diagnosis	Histology	History of Active Cancer	Prolon ged Anti Coagu Lation	PET SUV Uptake	Complications
Blanc et al. [43]	2011	1	Ddx of embolus	Left lower lobe PA	Yes	Tumor embolism	Follicular thyroid cancer	Yes	No	High	None
Chamorro et al. [44]	2012	1	Ddx of embolus	Right main PA	Yes	Tumor embolism	Primary retroperitoneal synovial sarcoma	Yes	Yes	High	None
Dhillon et al. [45]	2012	1	PET positive area without LNs	Hilar LN with incidental finding of endovascular lesion	No	Tumor embolism	Melanoma	Yes	yes	High	None
Dusemundet al. [46]	2013	1	Ddx of endovascular lesion	Left lower PA	Yes	Tumor embolism	Renal synovial sarcoma	Yes	No	Low	None
Schmid et al. [47]	2013	1	Ddx of embolus	Left lower lobe PA	Yes	Tumor embolism	Renal synovial sarcoma	Yes	Yes	Low	None
Lee et al. [48]	2014	1	Ddx of embolus	Right main PA	Yes	Tumor embolism	Hepatocellular carcinoma and rectal adenocarcinoma	Yes	No	N/A	None
Modi et al. [49]	2014	1	Ddx of endovascular lesion	Rightmain PA	Yes	Tumor embolism	Leiomyosarkoma	Yes	Yes	N/A	None
Desai et al. [50]	2015	1	Hilar nodes enlargment	Hilar soft tissue with endovascular lesion	Yes	Tumor embolism	Renal cell carcinoma	Yes	Yes	N/A	None
Vial et al. [51]	2015	1	Ddx of embolus	Left main PA	Yes	Tumor embolism	Colorectal cancer	Yes	Yes	High	None
Horowitz et al. [52]	2013	1	Left hilar mass surrounding and invading left pulmonary artery	Left main PA	Yes	Invasion from hilar mass	Poorly differentiated carcinoma	No	Yes	N/A	None
Dhamija et al. [53]	2012	1	Hilar nodes enlargment	Left PA	Yes	Invasion from hilar LN	Breast cancer	Yes	N/A	High	None
Lee et al. [54]	2016	1	Right paratracheal mass invading SVC	SVC	Yes	Invasion from paratracheal mass	Poorly differentiated adenocarcinoma	No	No	High	None
Park et al. [34]	2011	2	Ddx of endovascular lesion	1. Left main PA and left lower lobe PA 2. Left main PA	Yes	1. Vascular tumor 2. Thrombus	1. Pulmonary artery sarcoma 2. PE	No	No	1. High 2. Mild increase	None
Shingyogi et al. [55]	2013	1	Ddx of endovascular lesion	Bilateral PA (Biopsy from left lower PA)	Yes	Vascular tumor	Pulmonary artery sarcoma	No	No	High	None
Kamaleshwaran et al. [56]	2014	1	Ddx of endovascular lesion	Bilateral PA (Biopsy site not mentioned)	Yes	Vascular tumor	Pulmonary artery sarcoma	No	Yes	High	None
Chan et al. [57]	2014	1	Ddx of endovascular lesion	Right main PA extending to right upper and right lower branches	Yes	Vascular tumor	Pulmonary artery sarcoma	No	No	N/A	None
Caraway et al. [58]	2015	1	Ddx of endovascular lesion	Right main PA	Yes	Vascular tumor	Pulmonary artery sarcoma	Yes (3 cancers)	No	High	None
Alsaid et al. [59]	2020	1	Ddx of endovascular lesion	Left main PA	Yes	Vascular tumor	Pulmonary artery sarcoma	No	No	N/A	None
Zhang et al. [60]	2021	4	Ddx of endovascular lesion	N/A	Yes	Vascular tumor	Pulmonary artery sarcoma	No	N/A	N/A	1 death
Pott et al. [61]	2022	1	Ddx of pulmonary hypertension	Left main PA	Yes	Vascular tumor	Pulmonary artery sarcoma	No	Yes	High	None
Owen et al. [62]	2016	1	Ddx of endovascular lesion	Left main PA	Yes	Vascular tumor	Inflammatory myofibroblastic tumour	No	No	High	None
Qian et al. [63]	2021	1	Ddx of endovascular lesion	Left main and left upper PA	Yes	Septic embolus	Septic embolus	No	No	N/A	None

N/A: non-applicable.

Most cases of tumor embolism were initially misdiagnosed as PE and treated with anticoagulation for a prolonged period. Lack of resolution of the filling defect despite adequate treatment was the main rationale based on which the suspicion of a tumor embolism arose. However, it is suggested that in more than half of the PEs, a residual thrombus is detected in follow up at 6 months [29]. Given that a non-resolving thrombus is not always a tumor embolism, sometimes a histological confirmation may be needed.

PET scan has also been studied as a potential tool for differentiation of filling defects between thrombi and tumor emboli, but no specific accuracy has been reported. Based on the cases described in Table 2, no strong hypothesis favoring PET can be made. It is also noteworthy that the vast majority of the patients were already diagnosed with malignancy, which is related both with PE and tumor emboli. While PET imaging may indicate increased metabolic activity, histological confirmation through EBUS-TBNA remains crucial for definitive diagnosis in cases of a non-resolving embolic lesion despite adequate treatment.

In all but one case in the literature, the EBUS-TBNA intravascular approach was safe with no complications.

### 3.3. Vascular Tumors

Primary vascular tumors are exceedingly rare and carry a poor prognosis. Due to their rarity, few data exist on their diagnosis and management. Usually they are identified during surgery or in autopsies and high awareness is warranted for timely diagnosis.

EBUS-TBNA has been helpful in diagnosing these kinds of tumors. Eleven cases have been reported of primary pulmonary artery sarcoma (PAS) [34,55,56,57,58,59,60,61] and a case of inflammatory myofibroblastic tumor [62] diagnosed with EBUS-TBNA intravascularly. Contrary to what was reported for tumor embolism, most of these cases were not considered initially as possible PEs and no anticoagulation was administered, as shown in Table 2. In all the cases that PET findings were available, there was a high SUV uptake. The main indication for EBUS in those cases was to diagnose a suspicious tumor for pulmonary artery sarcoma, based on a low-attenuation filling defect on CT and extravascular extension of the lesion.

Zhang et al. documented four patients diagnosed with pulmonary artery sarcoma with EBUS [60]. In this series, a patient died during the procedure due to suspected vascular complications. Interestingly, no active or major bleeding was visualized after a biopsy was taken and the authors hypothesized that a vasospasm of the pulmonary artery or a clot formed after aspiration fully obstructed the vessel and lead to death. Questions were raised on the safety of EBUS-TBNA for the diagnosis of endovascular lesions, but the rest of the literature reports no complications known up to now.

We have reported a case of a 34-year-old male, nonsmoker, who presented in our Interventional Pulmonology Unit with hemoptysis, dyspnea, and an endovascular tumor of the left PA [59]. Intravascular EBUS-TBNA was performed, and the biopsy revealed an intimal sarcoma of the PA without any complications. Subsequently, the patient underwent surgical curative treatment.

Timely diagnosis is crucial as survival is poor for PAS and complications occurring due to an untreated PE or a wrongly treated tumor embolism can be devastating. The treatment modality associated with longest survival in PAS is surgical excision, a procedure which should be made with caution. Intravascular TBNA seems safe and feasible at first sight. Larger and prospective studies are warranted to prove this though.

### 3.4. Infectious Involvement

Qin et al. [63] described a case of septic embolism in a patient with bacteremia and a left hilar mass extending to the left main and left upper lobe PA. EBUS-TBNA was performed in the endovascular lesion and revealed the septic embolus. After antimicrobial treatment the lesion nearly disappeared, demonstrating the diagnostic value of EBUS in such scenarios. Other potential infectious diseases affecting the thoracic vasculature in immunocompromised patients include fungal infections like Aspergillus sp. and could potentially be successfully assessed by EBUS. There is a case of pulmonary artery obstruction due to echinococcosis diagnosed with EBUS imaging (visualization of cysts) [64].

## 4. T4 Assessment

Accurate staging is critical for determining prognosis and treatment strategies. EBUS and EUS-B are the recommended staging processes for non-small cell lung cancer (NSCLC) when it comes to mediastinum. However, endoscopic T4 staging by the same procedures is increasingly performed. Among other T4 criteria, invasion of great vessels or the heart can be observed with endosonography [65]. CT and PET are neither adequately sensitive nor specific for T4 assessment, particularly as far as vascular invasion is concerned.

To our knowledge, the first case report, of T4 diagnosis due to vessel invasion depicted with EBUS was by MacEachern et al. [66] in 2008. In this case, the right pulmonary artery and vein invasion by a right upper lobe mass was shown.

Alici et al. evaluated EBUS efficacy for detecting mediastinal or hilar vascular invasion [67]. Vascular invasion was defined as visualization of the tumor tissue within the vessel lumen and loss of vessel–tumor hyperechoic interface. Retrospectively, 55 cases were reviewed and the agreement between CT and EBUS was examined. The most involved vessels were the left and right main pulmonary arteries and the right superior pulmonary vein. The intra-agreement of both modalities in detecting vascular invasion was poor (κ = 0.268, *p* = 0.028). Surgery and thus histological data confirmation were available in only 9/55 patients. For those nine cases, sensitivity, specificity, positive and negative predictive values, and accuracy were encouraging in favor of EBUS over CT, but no gold standard to compare all the cases was readily available. Due to its retrospective nature, full assessment for T4 status was not guaranteed.

Kuijvenhoven et al., in a multicenter retrospective study, evaluated 104 patients with a primary lung tumor and subsequent surgical-pathological staging for T4, using EBUS in comparison to CT [68]. T4 status was defined as a tumor invading the diaphragm, mediastinum, heart, great vessels, trachea, recurrent laryngeal nerve, esophagus, vertebral body, or carina. Histologically, 35% of the patients had T4 status according to EBUS, based on vascular (*n* = 17), mediastinal (*n* = 15), both vascular and mediastinal (*n* = 3), or esophageal invasion (*n* = 1). With EBUS, twenty-three true positive cases were detected: mediastinal invasion (*n* = 12) or vascular invasion (*n* = 11), pulmonary artery (*n* = 9), pulmonary vein (*n* = 1) or azygos vein (*n* = 1), five false positive cases: mediastinal invasion (*n* = 1), pulmonary artery invasion (*n* = 3), or pericardium invasion (*n* = 1), and thirteen false negative cases: mediastinal invasion (*n* = 3), vascular invasion (*n* = 9), both mediastinal and vascular invasion (*n* = 1). Sensitivity, specificity, PPV, and NPV of EBUS for diagnosing T4-status were 63.9%, 92.6%, 82.1%, and 82.9%, respectively. While the sensitivity, specificity, PPV, and NPV of combined CT/EBUS for diagnosing T4-status were 90.9%, 77.3%, 66.7%, and 94.4%, respectively. The patients were evaluated for combined CT/EBUS characteristics as long as the two exams were concordant. Neither EBUS nor CT alone seem accurate enough for assessing T4-status, but their combination could increase the negative predictive value. Unfortunately, the bronchoscopist were not blinded for T4 status according to CT and thus a parameter of bias might be present.

There is also a previous retrospective clinical study by Kuijvenhoven et al. [69] assessing the efficacy of esophageal ultrasound (EUS) to detect the T4 status in NSCLC, including three cases of EUS-b among the total of seventy-four cases. The authors conclude that EUS along with CT improves significantly loco-regional staging.

While EBUS alone is insufficient for definite T4 staging as visualization of the whole vasculature of the lung is not feasible, its integration with CT seems to significantly increase the diagnostic accuracy. The ability to identify subtle changes such as loss of the hyperechoic interface between the tumor and the vessel is the great advantage of EBUS and EUS. Although there are not enough data to support whether a sole EBUS indication is enough to definitely stage the patient.

**Table 3:** Reported cases of T4 vascular assessment with EBUS.

Author	Year	N. of Cases	Vessel Invasion	EBUS Compared to Surgical Confirmation	CT Compared to Surgical Confirmation	Combined EBUS/CT Compared to Surgical Confirmation	EBUS and CT Intra-Agreement
MacEachern et al. [66]	2008	1	Right PA and vein invasion	N/A	N/A	N/A	N/A
Alici et al. [67]	2007	55	PAs, Pulmonary veins, axygus, aorta, SVC, left atrium	9/55 patients: Sensitivity 100%, Specificity 75, PPV 100%, NPV 77.7%	Sensitivity 66.6, Specificity 33.3%, PPV 33.3%, NPV 55.5%	N/A	k = 0.268 (*p* = 0.028)
Kuijenhoven et al. [68]	2017	74 (EUS)	N/A	Sensitivity 42%, Specificity 95%, PPV 73%, NPV 83%	Sensitivity 76%, Specificity 61%, PPV 41%, NPV 88%	Sensitivity 83%, Specificity 100%, PPV 100%, NPV 97%	N/A
Kuijenhoven et al. [69]	2021	104	N/A	Sensitivity 63.9%, Specificity 92.6%, PPV 82.1%, NPV 82.9%	Sensitivity 61.5%, Specificity 37%, PPV 35.6%, NPV 63%	Sensitivity 90.9%, Specificity 77.3%, PPV 66.7%, NPV 94.4%	N/A

N/A: not applicable, PPV: positive predictive value, NPV: negative predictive value.

## 5. Transvascular Biopsy

EBUS-guided transvascular needle aspiration (TVNA) represents a significant advancement, enabling tissue sampling of lesions located behind major vessels. Up until recently, accidental puncture of a vessel was considered a dreaded event, although serious hemorrhagic complications (hematoma, hemomediastinum, or active bleeding) were very rarely reported. TVNA has been shown to be both feasible and safe when performed by experienced operators [70]. More often EBUS-TVNA may be considered when lesion or lymph nodes lay behind major vessels, which in the literature seems to be needed in only 1–2% of the time [70,71].

Potential complications of TVNA could include endobronchial, mediastinal, retroperitoneal or peritoneal bleeding, hematomas formation, dissection of the vessel or dislocation of an atherosclerotic plaque, as well as hematogenous tumor seeding. However, reports of such dreadful complications are extremely rare. High-pressure vessels seem not to evoke major bleeding due to the elastic properties of their thicker muscular wall, while in the low-pressure vessels bleeding may be self-limited and standard hemostasis measures seem to be enough. However, a transvascular approach should only be performed by experts when other diagnostic means of minimally invasive diagnostic procedures have been exhausted.

The first intentional TBNA through the PA was reported by Vincent et al. [72] in 2006 in a patient with a carcinoid tumor adjacent to the left inferior PA not otherwise approachable. No complications were reported. During the curative pneumonectomy which followed, the puncture site could be observed without hematoma or bleeding.

Since then, many reports of transpassing the PA by EBUS have been reported [73,74,75,76,77,78,79,80,81,82,83]. Boujaouade et al. [73] had two cases of successful and uncomplicated transvascular approaches (right PA). Panchabhai et al. [71] reported 10 more cases of EBUS-TVNA puncturing pulmonary arteries and their branches with the same rates (9/10 diagnostic and no complications). Folch et al. [74] reported 10 cases of lymph node 5 station EBUS-TBNA through the left PA with a diagnostic yield of 90% and no complications. Perathur et al. [79] reported a case series of eight patients in whom ROSE (rapid on-site evaluation) was performed for EBUS-TVNA. From one to three passes per EBUS-TVNA were needed and the diagnostic yield was 87.5%. No complications occurred whatsoever.

Botana Rial et al. [84] reported accidental puncture of PA which caused intramural hematoma and hemomediastinum, while Torky et al. [85] observed mediastinal hematoma following accidental puncture of an abnormally located bronchial artery. No other major complications have been reported from the accidental puncture of vessels during EBUS-TBNA.

Transaortic with EBUS was first reported by Wallace et al. [86]. Combined EBUS and EUS were performed in a patient with a mass adjacent to the descending thoracic aorta. An uncomplicated puncture was performed through the aorta during EUS which proved to be diagnostic for NSCLC.

Von Bartheld et al. [87] assessed the safety of transaortic EUS-guided FNA for paraortic lung tumors and lymph nodes. These lesions can easily be identified by esophageal EUS, as the fluid containing aorta is an ideal medium for ultrasound waves. A total of 14 patients with an indication for biopsy of lesions not reachable otherwise were recruited. EUS-guided FNA was diagnostic for malignancy in 64% patients and for the rest was inconclusive. It is worth noting that only a single puncture was performed, a fact that maybe explains the lower-than-expected diagnostic yield from such procedures. In two patients, a local hematoma was visible via the ultrasound immediately after the puncture. Ravaglia et al. [88] also reported in favor of safety and efficacy of transaortic approach (Table 3).

Additionally to the above there is a report of a successful of EUS-B TVNA by Tamburrini et al. [89] through the left subclavian artery.

**Table 4:** Reported cases of EBUS (or EUS or EUS-B) transvascular needle aspiration (TVNA).

Author	Year	N. of Cases	Location of Target Lesion	Which Vessel Was Transversed	Diagnosis Achieved	Complications
Vincent et al. [72]	2006	1	Left hilar mass adjacent to left inferior pulmonary artery	Left inferior PA (EBUS)	Carcinoid tumor	None
Wallace et al. [86]	2007	1	Mass in left upper lobe adjacent to the descending aorta	Descending aorta (EUS)	NSCLC	None
vonBartheld et al. [87]	2009	14	9 left lung mass, 5 LN5 or LN6	Aorta (EUS)	Malignancy (9/14 patients—64% definitive diagnosis), non-diagnostic (5/14 patients)	2 paraortic hematomas (viewed with US)
Boujaouade et al. [73]	2013	2	1. 11R, 2. Right perihilar lung nodule	1. Right PA, 2. Right Lower branch of PA (EBUS)	1. Lung adenocarcinoma, 2. Lung papillary adenocarcinoma	None
Cubero et al. [81]	2015	1	Left upper lobe nodule	Left pulmonary artery	Metastatic rectal adenocarcinoma	None
Panchabhai et al. [71]	2015	10	3 left hilar/parenchymal lesions, 3 LN5, 2 10L, 1 12L, 1 right middle lobe mass	PA and its branches (EBUS)	Malignancy (7/10), benign (2/10), non-diagnostic (1/10). 90% definitive diagnosis	None
Folch et al. [74]	2016	10	LN 5	Left PA (EBUS)	Malignancy (9/10), non-diagnostic (1/10)	None
Kazakov et al. [75]	2017	33	15 mediastinal node/mass, 10 lung mass, 7 hilar node/mass, 1 paravertebral mass	14 through PA, 19 through aorta—(14 EBUS, 19 EUS)	Malignancy (16/33), benign (2/33), false negative (6/33), 71% sensitivity	Minor hematomas (viewed with US)—not mentioned how many
Mehta A et al. [82]	2018	1	Lesion at 12R station	Right inferior PA (EBUS)	Spindle cell neuroendocrine neoplasm	None
Mehta R et al. [76]	2018	7	4 LNs, 2 masses, 1 cyst	Left main PA (EBUS)	Malignancy (2/7), bening (5/7)—Tissue adequacy 10/10	None
Ravagliaet al. [88]	2018	11	LN 5 and LN6	Aorta (with EUS)	Malignancy (4/11), non-diagnostic (7/11)—57% sensitivity, 45% accuracy	None
Cetinkaya et al. [80]	2018	4	4 Left hilar lesions	Left PA (EBUS)	Malignancy (2/4), benign (1/4), non-diagnostic (1/4)	None
Guedes etal [77]	2019	1	11L	Left PA (EBUS)	Squamous cell carcinoma	None
Molina et al. [70]	2019	100	49 LN5 (EUS), 8 LN6 (3 EBUS, 5 EUS), 21 lung lesions (15 EBUS, 6 EUS), 16 hilar node/mass (EBUS), 3 mediastinal mass (1 EBUS, 2 EUS), 1 adrenal nodule (EUS), 1 supraclavicular nodule (EUS), 1 hepatic hilum node (EUS)	58 through aorta (EUS), 32 PA (EBUS), 6 left subclavian artery (2 EBUS, 4 EUS), 2 portal vein (EUS), 1 jugular vein (EUS), 1 innominate vein (EBUS)	80/100 diagnostic (malignancy 62/100), non-diagnostic (20/100)	1 aortic pseudoaneurysm
Tamburini et al. [89]	2019	1	Left upper lobe lesion	left subclavian artery (EUS-B)	Adenocarcinoma	None
Naaman et al. [78]	2021	35	Ν/A	29 main PA and its branches, 3 azygus vein, 2 brachiocephalic vein, 1 SVC	Diagnostic yield 95.6% for malignancy, 88.6% overall	4/35 (11.4%): hemoptysis, AECOPD, rapid rate on top of atrial fibrillation
Perathur et al. [79]	2021	8	4 left lung mass, 3 right hilar mass, 1 LN5	3 Interlobar PA, 5 Left main PA	Diagnostic yield 87.5% (4 adenocarcinoma, 2 tuberculosis, 1 esophageal cancer, 1 Spindle Cell Neuroendocrine cancer	4/8 (50%): mild bleeding during bronchoscopy
De Vega Sanchez et al. [83]	2021	1	11R	Right PA branch	Uterine Leiomyosarcoma	None

Nevertheless, transversing the aorta is not new. Transaortic celiac plexus block [90] and punctures for translumbar aortography have been reported only followed by not life-threatening local hematomas [91].

The largest series of TVNA by Molina et al. [70] reported 100 patients retrospectively. Aorta, PA, left subclavian artery, portal vein, and innominate vein were transpasssed with low complication rates (aortic pseudoaneurysm was reported in one patient). Multivariate analysis showed that independent predictors of inadequate sampling material during TVNA were as follows: traversing of the aorta, targets smaller than 10 mm, and the need for three or more passes.

In two recent metanalysis by Yang et al. and Giri et al. [92,93] TVNA was found to have a sample adequacy of 91.5%, an overall diagnostic yield of 82.1–85%, and a major complication rate of 1.4–2.71%. The complications reported were bleeding, pseudo-aneurysm of the aorta, hemoptysis, and acute hypoxic respiratory failure. The authors advise avoiding a fanning technique in such cases as well as to observe the area of puncture for a few minutes before withdrawing the endoscope. They conclude that EBUS/EUS-TVNA should only be considered and performed when it is anticipated that the results of TVNA will significantly impact the clinical management, and no alternative diagnostic approach is possible.

## 6. Conclusions

EBUS with color doppler enables localization of the vessels adjacent to the bronchi and pulmonologists become more and more familiar with the pulmonary vasculature.

PE in cancer patients can be diagnosed and followed sufficiently while T4 staging assessment has shown to be effective with regards to major vessels and heart invasion. Future addition of vascular assessment in the methodology of Lung Ca staging will require the development of a protocol and a specific training curriculum. EBUS/b-EUS TVNA has shown a favorable safety profile, but a number of unanswered clinical questions have to be addressed in randomized studies before clear guidelines can be outlined. The proper selection of patients and bronchoscopist’s expertise are critical. Limitations remain in this approach such as safety, and diagnostic yield vary based on operator variability and access to resources. Prospective randomized studies are needed to strengthen the feasibility and accuracy of the vasculature assessment in everyday practice.

Respiratory endosonography expands rapidly. The exploration of the vasculature, although not yet been systematically and fully evaluated, might prove itself a game changer in the management of our patients.
